# Enteric viral pathogens and child growth among under-five children: findings from South Asia and sub-Saharan Africa

**DOI:** 10.1038/s41598-024-64374-0

**Published:** 2024-06-15

**Authors:** Rina Das, Md. Ahshanul Haque, Karen L. Kotloff, Dilruba Nasrin, M. Jahangir Hossain, Dipika Sur, Tahmeed Ahmed, Myron M. Levine, Robert F. Breiman, A. S. G. Faruque, Matthew C. Freeman

**Affiliations:** 1 Gangarosa Department of Environmental Health, Rollins School of Public Health, Emory University, Atlanta, GA 30322 USA; 2 Nutrition Research Division, icddr,b, Dhaka, 1212 Bangladesh; 3 University of Maryland School of Medicine, Baltimore, MD USA; 4 Medical Research Council Unit the Gambia at the London School of Hygiene and Tropical Medicine, Fajara, The Gambia; 5 National Institute of Cholera and Enteric Diseases, Kolkata, West Bengal India; 6 James P. Grant School of Public Health, BRAC University, Dhaka, 1212 Bangladesh; 7 Department of Global Health, University of Washington, Seattle, WA 98104 USA; 8 Hubert Department of Global Health, Rollins School of Public Health, Emory University, Atlanta, GA 30322 USA

**Keywords:** Diseases, Gastroenterology, Medical research, Risk factors

## Abstract

Enteric viral pathogens are associated with a significant burden of childhood morbidity and mortality. We investigated the relationship between viral pathogens and child growth among under-5 children. We analyzed data from 5572/22,567 children enrolled in the Global Enteric Multicenter Study across seven study sites (2007–2011). Multiple linear regression was used to examine the association between the viral pathogens and changes of length/height-for-age (HAZ), weight-for-age (WAZ), and weight-for-length/height (WHZ) z-scores, stratified by diarrheal symptoms and adjusted for potential covariates. Rotavirus (18.51%) and norovirus (7.33%) were the most prevalent enteric viral pathogens among symptomatic and asymptomatic under-5 children, respectively. Infection with individual enteric viral pathogens hurts child growth in asymptomatic children. However, the relationship with HAZ was less clear and statistically non-significant. On the other hand, the combined viral pathogens demonstrated a strong negative influence on child growth [WAZ: β coef.: − 0.10 (95%, CI − 0.15, − 0.05); *P* < 0.001 and WHZ: β: − 0.12 (95% CI − 0.17, − 0.07); *P* < 0.001] among asymptomatic children. Infection with any viral pathogen was associated with growth shortfalls [HAZ: β: − 0.05 (95% CI − 0.09, 0.00); *P* = 0.03 and WAZ: β: − 0.11 (95% CI − 0.16, − 0.07); *P* < 0.001 and WHZ: β: − 0.13 (95% CI − 0.18, − 0.09); *P* < 0.001], though the relationship with HAZ was less evident and became statistically non-significant in older children. Notably, among symptomatic children with moderate-to-severe diarrhea, individual enteric viral pathogens, as well as the combined effects of these pathogens [WHZ: β: 0.07; (95% CI 0.01, 0.14); *P* = 0.03] and the presence of any virus [HAZ: β: 0.09 (95% CI 0.05, 0.13) & WAZ: β: 0.08 (95% CI 0.03, 0.12); *P* < 0.001], exhibited positive effects on child growth. While previous studies hypothesized that several viral pathogens had a conflicting controversial role in child growth, we find clear indications that enteric viral pathogens are associated with growth shortfalls, specifically among asymptomatic children. These findings highlight the need for preventive strategies targeting children with enteric viral pathogens, which could address the consequences of growth faltering.

## Introduction

There are, each year, approximately 1.7 billion cases of diarrhea^1^ which results in nearly 525,000 fatalities among children under the age of 5^1^. The highest rates of morbidity and mortality are among the poorest populations in sub-Saharan Africa and Southeast Asia^2^. Despite reducing mortality by a third over the past decade, diarrhea remains the second major cause of death among children under five, predominantly impacting those economically disadvantaged^3^. Beyond the diarrheal burden, the health impact of asymptomatic and chronic infections with viral, bacterial, and protozoal enteropathogens is not well- quantified^4^ and is likely significant.

Childhood malnutrition, associated with adverse long-term effects on learning capacity and work productivity, has been linked to the household economy, nutritional shortfalls, schooling of mothers, and childhood feeding practices^5,6^. The association between childhood diarrheal disease and child linear growth faltering in developing countries is well-described^7–9^. However, the impact attributed to specific enteric viral pathogens on child growth has not been quantified, and a limited set of viral pathogens has been examined. Many enteric viral pathogens are asymptomatic, and their presence in the gut is associated with an altered immune landscape that can have adverse effects on child health irrespective of diarrhea^10^. Efforts to address knowledge gaps concerning the impact of enteric viral pathogens on child growth are helping sustain the continuous reduction of child malnutrition unless global challenges impede advancements in LMICs (Low- and middle-income countries). Quantifying the impact of enteric viral pathogen infection on child growth, independent of diarrhea symptoms, would support public health prevention efforts, including vaccination and improvements to water and sanitation conditions, to reduce malnutrition.

To characterize the impact of enteric viral pathogens infection on child growth, we investigated the relationship between enteric viruses (rotavirus, norovirus, adenovirus, astrovirus, and sapovirus), and child growth among under-5 children in South Asia and sub-Saharan Africa. We conducted data analysis from the Global Enteric Multi-site Study (GEMS), which was a matched case–control study of moderate to severe diarrhea in children < 5 years old in four sites in sub-Saharan Africa and three sites in South Asia^11^. GEMS collected data from symptomatic diarrheal and asymptomatic healthy children on, child anthropometry, wealth, household density, and WASH facilities and practices. We analyzed diverse anthropometric indices reflecting enteric viral pathogens' long-term impact on child growth. To our knowledge, this is the first study to use laboratory-confirmed, children with symptomatic and asymptomatic enteric viral pathogens to analyze the association between enteric viruses and child anthropometric outcomes among children.

Our efforts were aimed at addressing crucial gaps in our understanding that have a substantial impact on the outcomes arising from enteric viral pathogens. These pathogens not only affect individuals with obvious diarrhoeal symptoms but also those with asymptomatic infections. These infections collectively lead to detrimental consequences for child growth and development. By addressing these gaps, we aimed to report the knowledge gaps that are largely playing leading roles in the aftereffects of enteric viral pathogens on both symptomatic and asymptomatic infections causing detrimental effects on child growth.

## Results

### Descriptive statistics and baseline demographics

**Figure 1:**
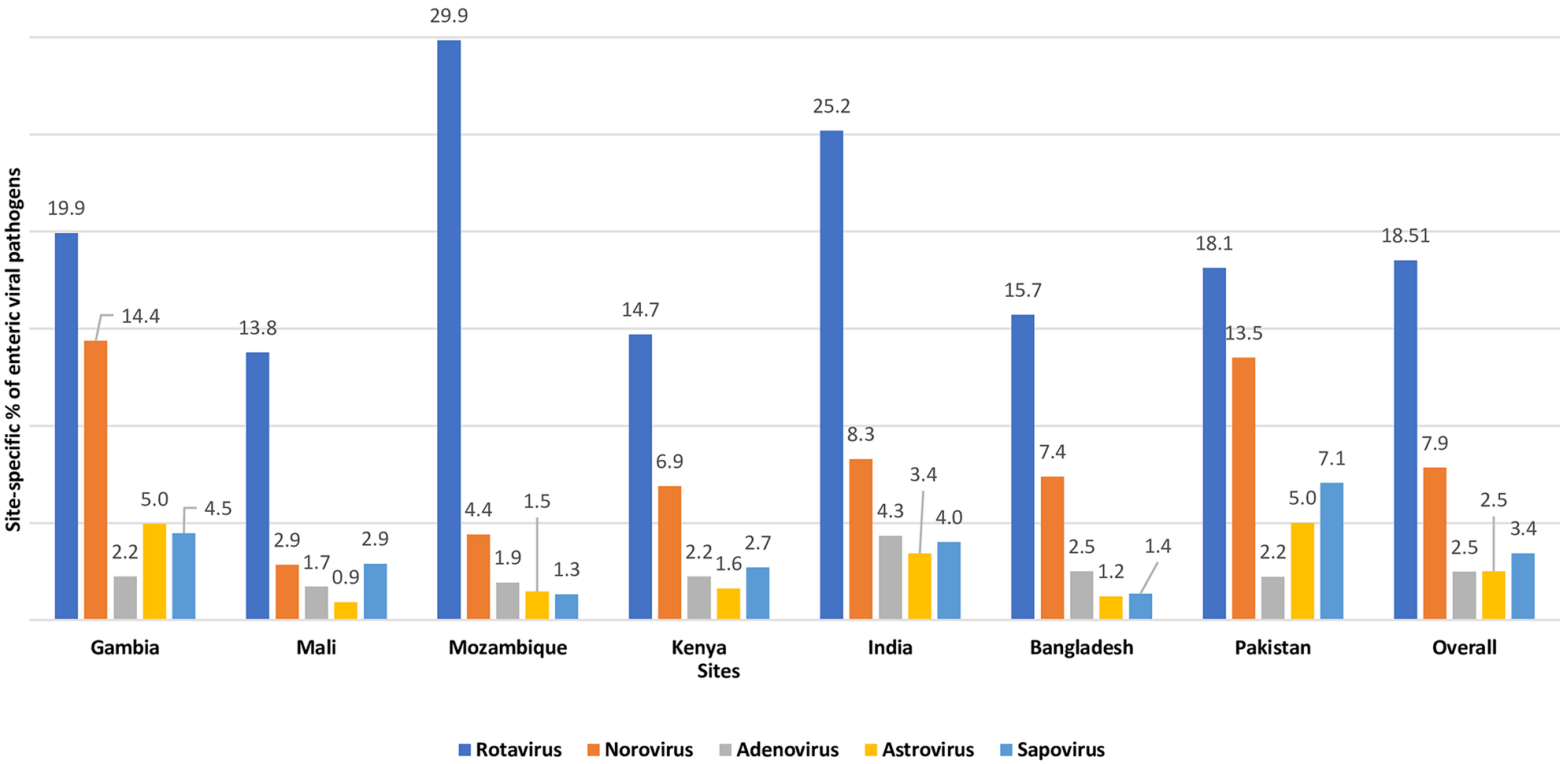
Site-Specific percentage of enteric viral pathogens isolated from the stool of under 5 children among the symptomatic MSD cases.

**Figure 2:**
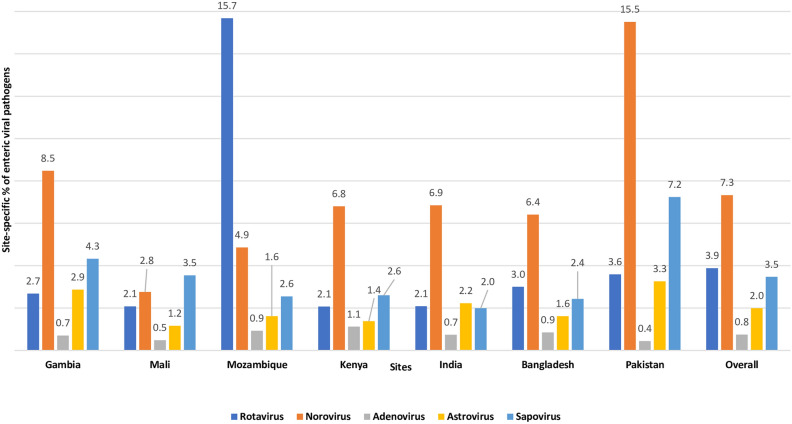
Site-Specific percentage of enteric viral pathogens isolated from the stool of under 5 children among the asymptomatic healthy controls.

**Figure 3:**
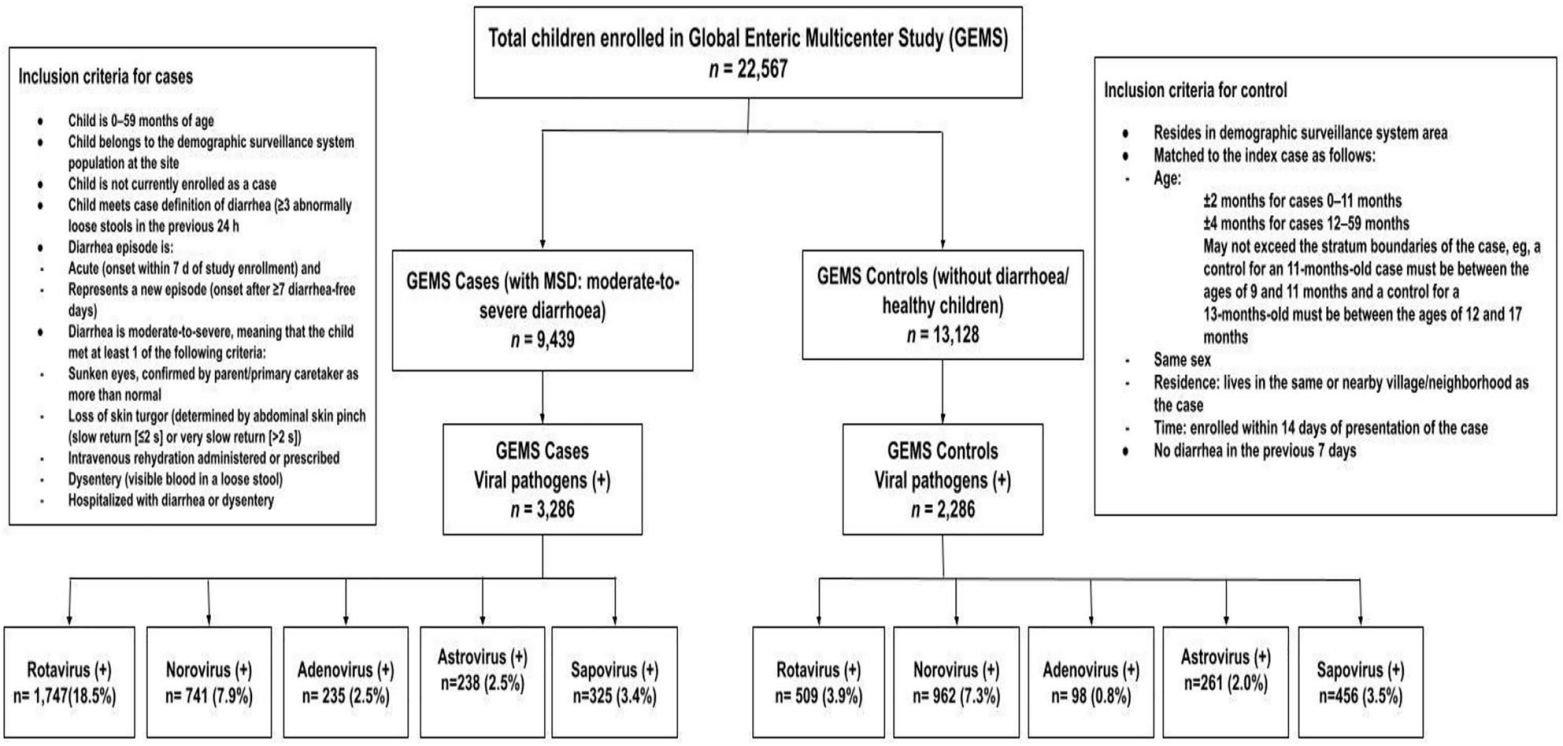
Study flow diagram.

Among the symptomatic diarrheal children, the detection of rotavirus was the highest in Mozambique (29.9%) and Indian (25.2%) study sites. In Gambia and Pakistan, norovirus was found in more than 13% of children. Astrovirus and sapovirus were found in less than 5% of all the sites (Fig. 1). Among the Asymptomatic children, the detection of rotavirus was highest in Mozambique (15.7%) and norovirus in Pakistan (15.5%) site (Fig. 2). Overall, in 7 sites, detection of rotavirus was 18.5% among the symptomatic MSD children and 3.9% in asymptomatic children, and detection of norovirus was 7.9% and 7.3% in symptomatic MSD children and asymptomatic children respectively (Fig. 3).

The baseline demographic characteristics of both symptomatic and asymptomatic children are presented in Table 1. For this analysis, we included 5572 children who were positive for enteric viral pathogens. Among them, 3286/9439 were symptomatic children with MSD and 2286/13,128 were asymptomatic children without diarrhea (Fig. 3).

**Table 1:** Baseline characteristics of the symptomatic MSD and asymptomatic children in South Asia and sub-Saharan Africa.

Characteristics n (%)	Asymptomatic children n = 13,128	Symptomatic children n = 9439	*P* value
Enteric viral pathogens			
Rotavirus	509(3.9)	1747 (18.5)	< 0.001
Norovirus	962(7.3)	741(7.9)	0.14
Adenovirus	98(0.8)	235(2.5)	< 0.001
Astrovirus	261(1.9)	238(2.5)	0.01
Sapovirus	456(3.5)	325(3.4)	0.90
Demographics			
Age group			
0–11 Months	4878 (37.2)	4030 (42.7)	< 0.001
12–23 Months	4381 (33.4)	3205 (33.9)	
24–59 Months	3870 (29.5)	2205 (23.4)	
Gender (girl)	5651 (43.0)	4095 (43.4)	0.61
Baseline anthropometry			
HAZ¥	− 1.34 ± 1.31	− 1.34 ± 1.37	
WAZ¥	− 1.08 ± 1.32	− 1.51 ± 1.39	
WHZ¥	− 0.47 ± 1.43	− 1.06 ± 1.49	
Breastfeeding status (age appropriate)			
Breastfeed	9039 (68.9)	6741 (71.4)	
Non-breastfeed	4090 (31.2)	2698 (28.6)	< 0.001
Wealth quintile			
Poorest	2510 (19.1)	2027 (21.5)	0.001
Lower middle	2590 (19.7)	1813 (19.2)	
Middle	2834 (21.6)	1993 (21.1)	
Upper middle	2522 (19.2)	1780 (18.9)	
Richest	2672 (20.4)	1821 (19.3)	
WASH			
The main source of drinking water			
Tube well water	2994 (22.8)	1675 (17.7)	< 0.001
Non-tube well water	10,135 (77.2)	7765(82.3)	
Toilet facility			
Sanitary/semi sanitary	12,289 (93.6)	8979 (95.1)	< 0.001
Non-sanitary	840 (6.4)	461 (4.9)	
Hand washing			
With soap and water	9762 (74.4)	7131 (75.6)	< 0.001
Without soap	3365 (25.6)	2308 (24.5)	
Clinical features			
Dysentery	–	2238 (23.7)	–
Fever	–	5849 (61.9)	–
Vomiting	–	3646 (38.6)	–

^¥^Mean ± SD (standard deviation); height/length-for-age, weight-for-age, and weight-for-height/ length z-scores (HAZ/LAZ, WAZ, and WHZ); Vomiting >  = 3 times per day.

Among the symptomatic children (n = 9439), rotavirus was detected in 1747 (18.5%), norovirus in 741 (7.9%), adenovirus in 235 (2.5%), astrovirus in 248 (2.5%), and sapovirus in 325 (3.4%) children (Table 1). All five enteric viral pathogens were more often (> 40%) detected among younger children (age 0–11 months), despite that more than 70% of children were reported (exclusively and partially) breastfed (age up to 24 months). Most of the children who were enteric viral pathogen positive used non-tube well water as the main source of drinking water (> 80%) in the household, more than 93% households used improved toilet facility (sanitary/semi sanitary), and more than 70% caregivers used water and soap for hand washing in comparison with the children who were negative for enteric viral pathogens (S1 Table).

Comparisons of demographic characteristics between virus positive and negative and stratified by diarrhea symptoms are found in S1 Table. Vomiting (66.3%) were more common (*P* value < 0.001) among the children with rotavirus-positive children than rotavirus-negative children. Dysentery was observed among 29% of children who were norovirus positive (S1 Table) than norovirus negative children (*P* value < 0.001).

Among the asymptomatic children, rotavirus was detected in 509 (3.9%), norovirus in 962 (7.3%), adenovirus in 98 (0.8%), astrovirus in 261 (1.9%), and sapovirus in 456 (3.5%) children (Table 1). All five enteric viral pathogens were more often (> 38%) detected among younger children (in age 0–11 months), and most of the children who were enteric viral pathogens positive used non-tube well water as main source of drinking water (> 80%); more than 90% of the household had improved toilet facility, almost 70% caregivers used water and soap for handwashing in comparison with the children negative for enteric viral pathogens (S1 Table).

### Association between enteric viral pathogens and child growth

Model 1: Any viral pathogens and child growth, stratified by diarrhea symptoms. Asymptomatic children who tested positive for any one of the enteric viral pathogens exhibited a decrease in child growth outcomes for HAZ (β: − 0.05; 95% CI − 0.09, 0.00), WAZ (β: − 0.11; 95% CI − 0.16, − 0.07), and WHZ (β: − 0.13; 95% CI − 0.18, − 0.09) for overall all children. Consistent associations were observed for WAZ and WHZ in the 0–11 months and 12–23 months age groups of asymptomatic children, but not for the older children. Conversely, symptomatic children with MSD, who tested positive for any one of the enteric viral pathogens showed increased child growth outcomes in overall all children and younger age groups (0–11 months and 12–23 months).

Model 2: Enteric viral pathogens and child growth, stratified by diarrhea symptoms. Rotavirus infection was negatively associated with WAZ (β coefficient: − 0.09; 95% CI − 0.17, − 0.01) and WHZ (β: − 0.10; 95% CI − 0.19, − 0.01) in asymptomatic children, a similar decreasing trend was observed in HAZ but statistically not significant. These findings remained consistent in younger age groups, with a negative association observed between rotaviral infection and HAZ (β: − 0.19; 95% CI − 0.31, − 0.07). Surprisingly, in children aged 24–59 months, asymptomatic rotavirus infection was associated with higher HAZ, WAZ, and WHZ. Among symptomatic children with MSD, rotavirus infection was found to have a positive impact on child growth outcomes overall and specifically in the age group of 0–11 months.

Norovirus infection was associated with a reduction in WAZ (β: − 0.12; 95% CI − 0.18, − 0.06) and WHZ (β: − 0.13; 95% CI − 0.19, − 0.06) among asymptomatic children, with consistent results observed in the 0–11 months age group. However, among symptomatic children, norovirus had the opposite effect, leading to an increase in child growth as indicated by higher WAZ, HAZ, and WHZ in overall and young children.

Adenovirus infection was associated with lower WAZ (β: − 0.28; 95% CI − 0.46, − 0.09) and WHZ (β: − 0.34; 95% CI − 0.54, − 0.14) among asymptomatic children, with these effects consistently observed in the younger age group. Notably, asymptomatic children aged 12–23 months positive for adenovirus also exhibited lower HAZ and WAZ. Surprisingly, adenovirus did not demonstrate any significant association with child growth in overall and all stratified groups among symptomatic children with moderate-to-severe diarrhea (MSD).

In asymptomatic children, no significant associations were observed between astrovirus infection and growth measures, as shown in Table 2. However, among symptomatic children with MSD at approximately 60-day follow-up, astrovirus infection was found to be correlated with lower HAZ (β: − 0.16; 95% CI − 0.28, − 0.03), but no significant associations were found with WAZ and WHZ. This trend was consistent for most age groups, except for children aged 12–23 months (Table 3).

**Table 2:** Association between enteric viral pathogens and child growth (Anthropometry: HAZ/LAZ, WAZ, and WHZ): results of multiple linear regression modeling (dependent variables—HAZ/LAZ, WAZ, and WHZ) among under-five children in South Asia and sub-Saharan Africa.

	Model 1*				Model 2^€^				Model 3 (combined viral pathogen)^¥^					
	Any viral pathogen		Rotavirus		Norovirus		Adenovirus		Astrovirus		Sapovirus		Combined viral pathogens	
	Coef. (95% CI) *	*P* value	Coef. (95% CI) *	*P* value	Coef. (95% CI) *	*P* value	Coef. (95% CI) *	*P* value	Coef. (95% CI) *	*P* value	Coef. (95% CI) *	*P* value	Coef. (95% CI) *	*P* value
Overall														
Asymptomatic children														
HAZ	− 0.05 (− 0.09, 0.00)	0.03	− 0.04 (− 0.12, 0.04)	0.34	− 0.05 (− 0.11, 0.01)	0.11	− 0.12 (− 0.31, 0.06)	0.19	− 0.04 (− 0.15, 0.08)	0.52	− 0.06 (− 0.15, 0.02)	0.16	− 0.04 (− 0.09, 0.01)	0.09
WAZ	− 0.11 (− 0.16, − 0.07)	< 0.001	− 0.09 (− 0.17, − 0.01)	0.03	− 0.12 (− 0.18, − 0.06)	< 0.001	− 0.28 (− 0.46, − 0.09)	0.003	0.02 (− 0.09, 0.14)	0.67	− 0.14 (− 0.23, − 0.05)	0.001	− 0.10 (− 0.15, − 0.05)	< 0.001
WHZ	− 0.13 (− 0.18, − 0.09)	< 0.001	− 0.10 (− 0.19, − 0.01)	0.03	− 0.13 (− 0.19, − 0.06)	< 0.001	− 0.34 (− 0.54, − 0.14)	0.001	0.04 (− 0.08, 0.17)	0.47	− 0.17 (− 0.26, − 0.08)	< 0.001	− 0.12 (− 0.17, − 0.07)	< 0.001
Symptomatic MSD children														
HAZ	0.09 (0.05, 0.13)	< 0.001	0.13 (0.08, 0.18)	< 0.001	0.1 (0.03, 0.17)	0.01	− 0.02 (− 0.14, 0.1)	0.74	− 0.16 (− 0.28, − 0.03)	0.01	0.05 (− 0.05, 0.15)	0.34	0.01 (− 0.05, 0.07)	0.74
WAZ	0.08 (0.03, 0.12)	< 0.001	0.07 (0.02, 0.12)	0.01	0.15 (0.08, 0.22)	< 0.001	0.04 (− 0.09, 0.16)	0.58	− 0.09 (− 0.22, 0.04)	0.17	0.15 (0.04, 0.26)	0.01	0.06 (0.0, 0.12)	0.06
WHZ	0.03 (− 0.02, 0.07)	0.24	− 0.01 (− 0.07, 0.04)	0.631	0.13 (0.05, 0.21)	0.002	0.05 (− 0.09, 0.18)	0.50	0.01 (− 0.14, 0.14)	0.99	0.17 (0.05, 0.28)	0.01	0.07 (0.01, 0.14)	0.03

*Model 1 and ^€^2: Adjusted for gender, breastfeeding status, primary caretaker’s education, WASH, wealth index, number of under 5 children in household, co-pathogens (ETEC, EAEC, *Shigella, Campylobacter*), site, and history of comorbidity (malaria, typhoid, pneumonia, diarrhea, dysentery) at day 60 follow up.

^¥^Model 3: The predictor variable titled “Enteric viral pathogens” was created for the combined effect of enteric viral pathogens as a composite indicator by principal component analysis (PCA) using all five enteric viruses: rotavirus, adenovirus, astrovirus, norovirus, and sapovirus.

Adjusted for gender, breastfeeding status, primary caretaker’s education, WASH, wealth index, number of under 5 children in household, co-pathogens (ETEC, EAEC, *Shigella*, *Campylobacter*), site, and history of comorbidity (malaria, typhoid, pneumonia, diarrhea, dysentery) at day 60 follow up.

Enteric viral pathogens were detected from the stool sample during enrollment; Anthropometric measurements were taken during enrollment and after 60 days of enrollment (during the follow-up visit); Separate models were performed to see the association of enteric viral pathogen.

*Coef.* β coefficient, *CI* confidence interval, *HAZ/LAZ* height/length-for-age, *WAZ* weight-for-age, *WHZ* weight-for-height z-scores.

**Table 3:** Association between enteric viral pathogens and child growth (Anthropometry: HAZ/LAZ, WAZ, and WHZ): results of multiple linear regression modeling (dependent variables—HAZ/LAZ, WAZ, and WHZ) among the different age groups in South Asia and sub-Saharan Africa.

	Model 1*				Model 2^€^				Model 3 (combined viral pathogens)^¥^					
	Any viral pathogen		Rotavirus		Norovirus		Adenovirus		Astrovirus		Sapovirus		Combined viral pathogens	
	Coef. (95% CI)*	*P* value	Coef. (95% CI)*	*P* value	Coef. (95% CI)*	*P* value	Coef. (95% CI)*	*P* value	Coef. (95% CI)*	*P* value	Coef. (95% CI)*	P value	Coef. (95% CI)*	*P* value
Symptomatic MSD children														
0–11 months														
HAZ	0.1 (0.04, 0.16)	0.001	0.12 (0.05, 0.19)	0.001	0.11 (0.01, 0.22)	0.03	0 (− 0.17, 0.16)	0.99	− 0.23 (− 0.4, − 0.07)	0.01	0.04 (− 0.12, 0.19)	0.64	0.01 (− 0.07, 0.1)	0.78
WAZ	0.08 (0.01, 0.15)	0.02	0.06 (− 0.02, 0.14)	0.124	0.22 (0.1, 0.34)	< 0.001	0.1 (− 0.09, 0.29)	0.28	− 0.2 (− 0.39, − 0.01)	0.04	0.18 (0, 0.36)	0.05	0.06 (− 0.04, 0.16)	0.23
WHZ	− 0.01 (− 0.08, 0.07)	0.86	− 0.05 (− 0.13, 0.04)	0.27	0.18 (0.05, 0.32)	0.01	0.11 (− 0.1, 0.32)	0.30	− 0.12 (− 0.33, 0.09)	0.27	0.18 (− 0.02, 0.38)	0.07	0.06 (− 0.05, 0.16)	0.33
12–23 months														
HAZ	0.11 (0.04, 0.19)	0.002	0.17 (0.08, 0.26)	< 0.001	0.08 (− 0.04, 0.2)	0.21	− 0.06 (− 0.26, 0.14)	0.56	0.05 (− 0.16, 0.26)	0.65	0.06 (− 0.11, 0.23)	0.47	0.03 (− 0.07, 0.12)	0.59
WAZ	0.12 (0.05, 0.2)	0.001	0.13 (0.04, 0.22)	0.004	0.12 (− 0.01, 0.25)	0.06	− 0.05 (− 0.26, 0.15)	0.60	0.13 (− 0.09, 0.35)	0.26	0.17 (− 0.01, 0.34)	0.06	0.09 (− 0.01, 0.19)	0.07
WHZ	0.09 (0.02, 0.17)	0.02	0.07 (− 0.02, 0.17)	0.142	0.12 (− 0.01, 0.25)	0.08	− 0.04 (− 0.26, 0.18)	0.73	0.15 (− 0.09, 0.38)	0.22	0.19 (0, 0.37)	0.05	0.11 (0, 0.22)	0.04
24−59 months														
HAZ	0.01 (− 0.08, 0.11)	0.77	0.06 (− 0.08, 0.21)	0.39	0.09 (− 0.06, 0.24)	0.25	0.07 (− 0.28, 0.42)	0.70	− 0.37 (− 0.67, − 0.07)	0.02	0.13 (− 0.11, 0.37)	0.29	− 0.04 (− 0.16, 0.09)	0.57
WAZ	0.02 (− 0.07, 0.11)	0.65	0.04 (− 0.1, 0.17)	0.60	0.08 (− 0.06, 0.21)	0.27	0.04 (− 0.28, 0.35)	0.81	− 0.24 (− 0.51, 0.03)	0.08	0.14 (− 0.07, 0.36)	0.19	0.01 (− 0.1, 0.12)	0.87
WHZ	0.03 (− 0.07, 0.12)	0.59	− 0.02 (− 0.16, 0.12)	0.78	0.05 (− 0.09, 0.19)	0.46	0.01 (− 0.32, 0.34)	0.96	− 0.01 (− 0.29, 0.26)	0.92	0.13 (− 0.09, 0.36)	0.25	0.07 (− 0.05, 0.19)	0.24
Asymptomatic children														
0–11 months														
HAZ	− 0.07 (− 0.14, 0)	0.06	− 0.19 (− 0.31, − 0.07)	0.003	− 0.06 (− 0.16, 0.04)	0.24	0.03 (− 0.27, 0.32)	0.86	0.02 (− 0.16, 0.21)	0.81	0.07 (− 0.08, 0.20)	0.38	− 0.01 (− 0.09, 0.07)	0.76
WAZ	− 0.19 (− 0.26, − 0.12)	< 0.001	− 0.22 (− 0.35, − 0.09)	0.001	− 0.22 (− 0.32, − 0.11)	< 0.001	− 0.31 (− 0.62, − 0.01)	0.05	− 0.02 (− 0.22, 0.18)	0.84	− 0.03 (− 0.18, 0.12)	0.67	− 0.15 (− 0.23, − 0.06)	0.001
WHZ	− 0.24 (− 0.32, − 0.16)	< 0.001	− 0.18 (− 0.33, − 0.04)	0.01	− 0.27 (− 0.39, − 0.15)	< 0.001	− 0.51 (− 0.85, − 0.16)	0.004	− 0.08 (− 0.30, 0.03)	0.46	− 0.14 (− 0.30, 0.03)	0.11	− 0.22 (− 0.32, − 0.12)	< 0.001
12–23 months														
HAZ	− 0.06 (− 0.13, 0.02)	0.14	0.01 (− 0.14, 0.15)	0.93	− 0.06 (− 0.16, 0.05)	0.29	− 0.33 (− 0.62, − 0.04)	0.02	− 0.23 (− 0.43, − 0.04)	0.02	− 0.05 (− 0.19, 0.10)	0.54	− 0.05 (− 0.14, 0.03)	0.21
WAZ	− 0.09 (− 0.17, − 0.02)	0.01	− 0.13 (− 0.28, 0.01)	0.08	− 0.07 (− 0.18, 0.03)	0.17	− 0.31 (− 0.61, − 0.02)	0.04	− 0.03 (− 0.24, 0.17)	0.75	− 0.12 (− 0.27, 0.02)	0.10	− 0.06 (− 0.14, 0.03)	0.19
WHZ	− 0.09 (− 0.17, − 0.01)	0.03	− 0.18 (− 0.34, − 0.02)	0.03	− 0.06 (− 0.17, 0.06)	0.33	− 0.22 (− 0.54, 0.10)	0.17	0.11 (− 0.11, 0.34)	0.32	− 0.15 (− 0.31, 0.01)	0.07	− 0.04 (− 0.13, 0.05)	0.37
24− 59 months														
HAZ	− 0.02 (− 0.1, 0.06)	0.62	0.23 (0.06, 0.4)	0.01	− 0.05 (− 0.16, 0.06)	0.36	0.02 (− 0.42, 0.42)	0.99	0.14 (− 0.05, 0.33)	0.14	− 0.34 (− 0.5, − 0.18)	< 0.001	− 0.09 (− 0.18, 0.00)	0.05
WAZ	− 0.04 (− 0.11, 0.03)	0.27	0.29 (0.13, 0.44)	< 0.001	− 0.07 (− 0.17, 0.03)	0.15	− 0.09 (− 0.47, 0.29)	0.64	0.16 (− 0.02, 0.33)	0.08	− 0.39 (− 0.54, − 0.25)	< 0.001	− 0.12 (− 0.2, − 0.04)	0.002
WHZ	− 0.04 (− 0.11, 0.04)	0.31	0.23 (0.07, 0.39)	0.01	− 0.05 (− 0.15, 0.05)	0.30	− 0.16 (− 0.54, 0.23)	0.43	0.11 (− 0.06, 0.28)	0.23	− 0.28 (− 0.43, − 0.13)	< 0.001	− 0.10 (− 0.18, − 0.02)	0.02

*Model 1 and ^€^2: Adjusted for gender, breastfeeding status, primary caretaker’s education, WASH, wealth index, number of under 5 children in household, co-pathogens (ETEC, EAEC, *Shigella, Campylobacter*), site, and history of comorbidity (malaria, typhoid, pneumonia, diarrhea, dysentery) at day 60 follow up.

^¥^Model 3: The predictor variable titled “Enteric viral pathogens” was created for the combined effect of enteric viral pathogens as a composite indicator by principal component analysis (PCA) using all five enteric viruses: rotavirus, adenovirus, astrovirus, norovirus, and sapovirus.

Adjusted for gender, breastfeeding status, primary caretaker’s education, WASH, wealth index, number of under 5 children in household, co-pathogens (ETEC, EAEC, *Shigella*, *Campylobacter*), site, and history of comorbidity (malaria, typhoid, pneumonia, diarrhea, dysentery) at day 60 follow up.

Enteric viral pathogens were detected from the stool sample during enrollment; Anthropometric measurements were taken during enrollment and after 60 days of enrollment (during the follow-up visit); Separate models were performed to see the association of enteric viral pathogen.

*Coef.* β coefficient, *CI* confidence interval, *HAZ/LAZ* height/length-for-age, *WAZ* weight-for-age, *WHZ* weight-for-height z-scores.

For children who tested positive for sapovirus, infection was associated with lower WAZ (β: − 0.14; 95% CI − 0.23, − 0.05) and WHZ (β: − 0.17; 95% CI − 0.26, − 0.08), and this pattern remained consistent among older children (aged 24–59 months), with lower HAZ, WAZ, and WHZ. However, sapovirus infection exhibited a different outcome in symptomatic children overall, showing an increase in child growth outcomes (Table 2).

Model 3: Composite measure of viral pathogens and child growth, stratified by diarrhea symptoms. The combined effect of viral pathogens was found to be associated with lower WAZ (β: − 0.10; 95% CI − 0.15, − 0.05) and WHZ (β: − 0.12; 95% CI − 0.17, − 0.07) among asymptomatic children. These associations were consistently observed in both the 0–11 months and 24–59 months of age of asymptomatic children (Table 3). However, interestingly, in the same model, increased child growth outcomes were observed for the combined viral pathogens in symptomatic MSD children.

Mixed-effect model: In our study, participants had two repeated measurements taken (during enrollment and at follow-up), and measurements within individuals are likely correlated. As a sensitivity analysis, we also analyzed our sample using mixed effects models as reported in Supplementary Tables 2–5 (S2 to S5 tables) to addresses the random within-participant fluctuation. We found results largely consistent with our original general linear models (Tables 2 & 3).

## Discussion

To our knowledge, this study is the first to investigate the relationship between enteric viral pathogens and child growth in children from birth to 5 years old, with and without diarrhea. We found evidence indicating that infection with individual enteric viral pathogens has an impact on child growth, specifically WAZ and WHZ, among asymptomatic children. These findings have important implications for addressing asymptomatic enteric viral pathogen infections in low-income settings. Among symptomatic children with moderate-to-severe diarrhea, individual enteric viral pathogens, as well as the combined effects of these pathogens and the presence of any virus, exhibited positive effects on child growth. Our findings suggest a need to reassess the decision-making process for children under 5 years old, considering the significant impact on growth and nutrition status. This highlights the importance of preventive measures and management strategies especially for asymptomatic viral infections in young children.

Our study found that asymptomatic young children who tested positive for viral pathogens, particularly rotavirus, had a higher prevalence of malnutrition. Among asymptomatic older children, rotavirus was associated with growth issues across all measures. Our findings support previous research indicating that young children are more susceptible to enteric virus infections, potentially due to their developing immune systems and reduced maternal antibodies after birth^12,13^. Additionally, at this age, the combination of supplementary food and breastfeeding often contributes to malnutrition and diarrhea in LMICs^12^. Infection with any viral pathogen was associated with growth shortfalls, though the relationship with HAZ was less evident and became statistically non-significant in older children. It is noteworthy that among symptomatic children with MSD, individual enteric viral pathogens, as well as the combined effects of these pathogens and the presence of any virus, exhibited positive effects on child growth. No significant associations were found between astrovirus infection in asymptomatic children and adenovirus infection in symptomatic children, possibly due to limited statistical power resulting from their low prevalence.

Previous studies conducted in similar settings have found links between childhood malnutrition and subclinical viral infections, but they did not measure viral burdens over time^14^. Our statistical models evaluated the impact of enteric viruses on child growth outcomes, considering different age groups among the asymptomatic children. Research has demonstrated that norovirus infection disrupts intestinal barrier function and impairs the role of tight junction proteins, resulting in the clinical symptoms of Environmental Enteric Dysfunction (EED)^15^. This condition, along with frequent episodes of diarrhea, could contribute to reduced growth by reducing the absorption capacity of nutrients and compromising intestinal barrier function. Furthermore, studies in LMICs have highlighted the poor quality of dietary proteins, leading to deficiencies in essential amino acids levels^16–18^. Consequently, it is possible that there was a decrease in the activity of the key growth regulation signaling pathway within cells, leading to reduced muscle and bone growth^19^. Another factor that could affect the severity of sapovirus infection in these situations is the variation in gut microbiota, which has been shown to either promote or resist the colonization of different enteric pathogens^20^. The impact of enteric pathogens on the composition of the gut microbiota is being studied, and it has been observed that certain changes in the microbiota can influence the susceptibility of hosts to other infections^20^. These factors may explain why viral pathogens affect the growth of asymptomatic children in our study.

Our analysis revealed a positive link between symptomatic enteric viral pathogens and child growth, possibly due to the administration of antibiotics during hospitalization for co-infections. The MAL-ED study identified *Shigella* and rotavirus as the primary causes of antibiotic treatment, accounting for 11.7% and 8.6% of diarrhea treatments, respectively^21^ due to their high burden and severity. Antibiotic treatment could act as a confounding factor when estimating the actual impact of enteric viral infections on child growth, as evidenced by similar findings in a systematic review and meta-analysis on *Campylobacter* infection^22^ and similar findings from the GEMS Bangladesh site^23^. A cross-sectional study conducted in Uganda found no significant association between rotaviral diarrhea and nutritional status^24^. Similarly, a study in Zambia observed a higher frequency of rotaviral diarrhea in children with better nutritional status^25^. Improved childhood nutrition and overnutrition, in conjunction with other manmade and climate-related factors, may play a significant role in the development of symptomatic rotavirus infections^26^. Malnutrition compromises the gut's integrity and weakens the normal immune system, thereby affecting the attachment and pathogenesis of rotavirus, which relies on a healthy epithelium for its action^27^. The preference of rotavirus for the nutritionally intact intestinal epithelium may contribute to the observed impact on anthropometric indices and higher prevalence of rotavirus infection. However, in our study, we did not find any association between any form of malnutrition and symptomatic MSD caused by viral pathogens, and the presence of severe malnutrition in our population limited our ability to determine if the association between viral pathogens and growth was influenced by nutritional deficits. As astrovirus is less common compared with other major gastroenteritis viruses, including norovirus and rotavirus^28^, it is an overlooked cause of diarrhea among vulnerable children worldwide. Surprisingly, in our analysis only symptomatic astrovirus infection causes poor linear growth. With the evidence presented here, we highlight the need for future research to explore the pathogenesis of astrovirus infection and child growth. The interpretation of the positive association between viral pathogens and growth outcome (WAZ and WHZ) among symptomatic MSD children may be affected using weight measurement sometimes taken at enrollment, four hours after rehydration, after additional rehydration if needed, and on leaving the health center at baseline.

Our study had limitations due to the lack of data on maternal factors, biomarkers, and had a short-term assessment with a single follow-up visit. However, we had a large, randomly sampled population, conducted high-quality lab procedures, and examined the effect of co-pathogens using multiple models. We investigated the association between enteric viral pathogens and growth faltering in children under five across seven sites, analyzing growth outcomes during the at-risk period (0–5 years). However, we couldn't establish a comprehensive framework for the biological transmission mechanism of enteric viral pathogens, nor establish a temporal association with the study outcomes due to the need for further longitudinal studies.

## Conclusion

Enteric viral pathogens, negatively affect growth in young children, even in the absence of diarrhea symptoms. Asymptomatic children experience growth shortfalls, while symptomatic children with astrovirus infection have poor linear growth. Future studies should investigate the impact of treating or preventing asymptomatic enteric viral infections on childhood malnutrition. It is important to include regular viral infection monitoring in nutritional assessments in endemic areas. Developing effective antiviral medications and vaccines for enteric viral pathogens is crucial for preventing childhood malnutrition.

## Methods

### Ethical considerations

This study did not require specific ethical approval as a secondary analysis of de-identified data. GEMS was approved by site-specific ethics committees and the University of Maryland School of Medicine^29^. The signed informed consent forms for the children’s participation in the study were collected from their parents/guardians (both diarrhea cases and healthy controls).

### Study design

GEMS was a prospective, age-stratified, matched case–control study that was carried out in seven study sites in South Asia (Bangladesh, India, and Pakistan) and Sub-Saharan Africa (The Gambia, Mali, Mozambique, and Kenya) between December 1, 2007, and March 3, 2011^11^. Cases were defined as under-5 children from the Demographic Surveillance System (DSS) catchment area who presented to the Sentinel Health Center within 7 days of the onset with a new and acute episode of moderate-to-severe diarrhea (MSD) (Fig. 3). Age, sex, and community-matched children without diarrhea in the previous 7 days, were randomly selected from the DSS database and were enrolled as controls (asymptomatic children) (Fig. 3). At the time of enrollment, a stool sample had been collected from each case and control. At the time of registration, nutritional assessments were conducted using the following criteria: weight, length/height, and mid-upper arm circumference (MUAC)^11^. GEMS field workers visited each enrolled child's home about 60 days following enrollment (acceptable range, 50–90 days)^11^. The anthropometry data and details of the comorbidities (dysentery, pneumonia, typhoid, malaria, and diarrhea) from those follow-up home visits were utilized in our analysis^30^.

### Outcome Variable

The primary measure of growth in our analyses was the height/length-for-age z-score (HAZ), weight-for-age z-score (WAZ), and weight-for-height z-score (WHZ). In our study, we used baseline (after rehydration in case of MSD) and endline HAZ, WAZ, and WHZ from enrolment to follow-up for the rotavirus, norovirus, adenovirus, astrovirus, and sapovirus positive children enrolled in GEMS^30^ (Fig. 3).

### Variables of interest

#### Enteric viral pathogens

Commonly found five enteric viruses: rotavirus, norovirus, adenovirus, astrovirus, and sapovirus were assessed in this study.

#### Specimen collection and laboratory procedure

The GEMS protocol incorporated Enzyme immunoassays which were performed to detect the viral pathogens. Rotavirus and adenovirus were detected using stool immunoassays (ELISA) and norovirus, astrovirus, and sapovirus by multiplex reverse transcriptase (RT)-PCR, available commercially and according to the manufacturer's protocols^31^, described elsewhere^31^.

#### Anthropometry

Height and weight were measured at enrollment and the 60-day follow-up visit for each child, and details of measuring methods were described elsewhere^29,30^. Using the WHO Child Growth Standards as the reference population, the HAZ/LAZ, WAZ, and WHZ were measured using a WHO SAS macro^11,30^.

#### Moderate-to-severe diarrhea (MSD)

MSD was defined as new and acute diarrhea (≥ 3 abnormally loose stools within the past 24 h) that started within the previous 7 days following at least 7 diarrhea-free days, with at least one of the following criteria for MSD: dehydration based on the study clinician’s assessment (sunken eyes; decreased skin turgor; or intravenous rehydration administered or prescribed); dysentery (visible blood in stools reported by the mother or observed by the study team); or hospitalization with diarrhea or dysentery^11,30^ (Fig. 3).

#### Vomiting, fever, and dysentery

Vomiting 3 or more times per day, and fever (at least 38°C and parental perception) determined by recall and dysentery (visible blood in stools) assessed by the attending clinician^11,30^.

#### Breastfeeding

Breastfed referred to both exclusive and partially breastfeed children under 2^30^.

#### Sociodemographic information

Included data about the child’s household including primary caretaker’s education (illiterate/ literate), and household size (number of children < 5 years of age), were considered explanatory variables. Households were categorized based on the wealth quintiles as socioeconomic status (SES) (poor, lower-middle, middle, upper-middle, and rich)^30,32^ by using principal component analysis. The survey includes data on asset indicators that can be grouped into three types: household ownership of consumer durables (clock/watch, bicycle, radio, television, bicycle, sewing machine, refrigerator, car); characteristics of the household’s dwelling (sanitation, main source of drinking water, rooms in the dwelling, building materials used, and electricity and cooking fuel); and household land ownership^30^. Children were sorted by the asset index and established cutoff values for percentiles of the population. Then the households were assigned to a group based on their value on the index. For expository convenience, they refer to the bottom 20% as “poorest,” the next 20% as “lower middle”, the next 20% as “middle”, the next 20% as “upper middle” and the top 20% as “richest,” but this classification does not follow any of the usual definitions of poverty^32^. Variables addressed WASH were the main source of drinking water (tube well water/ non-tube well water); sanitation facilities (improved toilet facility for disposal of human fecal waste available: sanitary and or semi-sanitary/non-sanitary), and the use of handwashing materials (water with soap/ without soap)^30^.

### Statistical analysis

#### Summary

We modeled the relationship between the presence of any enteric viral pathogen at baseline and the change in the child’s HAZ, WAZ, and WHZ in the subsequent ~ 60 days (model 1) and for each individual viral pathogen, controlling for all others (model 2). We also modeled the impact of a composite score (model 3) related to viral infection on child growth. All models were stratified by diarrhea symptoms, to account for the study design. We reported the child and household-level characteristics by using mean and standard deviation for continuous variables and frequency as a percentage for categorical variables to summarize the data.

#### Model 1

Modeling any viral pathogen. We used a generalized linear model. The main explanatory variable was the presence of intestinal viral pathogens, and the outcome variable was changes from enrollment and 60 days follow-up anthropometry (HAZ, WAZ, and WHZ); the anthropometry was taken in two-time points (on enrollment = 0 and day ~ 60 follow up = 1). Variables which were adjusted include age, gender, breastfeeding status, primary caretaker’s education, number of under-5 children at the house, WASH, co-pathogens (Enterotoxigenic *E. coli*, Enteroagressive *E. coli*, *Shigella*, and *Campylobacter*), comorbidity, time (since it was a repeated measured data, we adjusted the variable [time: 0 and 1] as co-variate); and study sites within the two regions, suggesting the association with the outcome as indicated in the literature were chosen for multivariable modeling. Separate models were constructed to assess the association of each enteric vital pathogen infection with a child’s HAZ, WAZ, and WHZ for symptomatic and asymptomatic infections.

#### Model 2

Adjusted model for all viral pathogens concurrently. We did similar things to model 2 as in model 1.

#### Model 3

Modeling a composite measure of viral infection. The predictor variable titled “composite enteric viral pathogen” was created for the combined effect of enteric viral pathogens as a composite indicator by principal component analysis (PCA) using all five enteric viruses. Our focus was on investigating the relationship between the enteric viral pathogens, specifically rotavirus, norovirus, adenovirus, astrovirus, and sapovirus with child growth. These viruses were analyzed together to identify any potential correlations among them. To create a composite indicator that represents the overall behavior of these viruses, for a dimension-reducing technique Principal Component Analysis (PCA) was employed. PCA is a statistical method used to transform a set of correlated variables into a smaller set of uncorrelated variables, called principal components. The first principal component obtained from PCA captures the maximum variance in the original data and represents a combination of the original variables in a way that best summarizes their shared information. The Kaiser–Meyer–Olkin (KMO) test was used to evaluate the sampling adequacy for performing PCA on the data. The KMO test assesses whether the data is suitable for PCA by measuring the proportion of variance among the variables that might be common variance. It determines the strength of correlations between variables and indicates whether the data is appropriate for dimension reduction using PCA. Generally, if the KMO value is greater than 0.5 (or 50%), it is considered acceptable for PCA. A higher KMO value indicates a better correlation among the variables and thus a higher adequacy for PCA. The KMO value can range from 0 to 1, with values closer to 1 indicating better suitability for PCA^33^. In our analysis, the KMO measure of sampling adequacy was 51%.

After conducting the PCA analysis, the first principal component was selected as the composite indicator. This component represents a linear combination of the original five viruses and is considered a synthetic variable that summarizes the common variance among them. By using the first principal component as the composite indicator, the study aims to capture the collective behavior of these enteric viral pathogens, making it easier to analyze and interpret their overall impact or relationship. In our data analysis, the first PCA explained the five measurable variables by 55.1%. We hypothesized that PCA would result in viral pathogen composites with loadings distributed across influential measures, as PCA aims to maximize explained variance using interrelationships between all available inputs, rather than concentrating weights in a single dominant virus. Therefore, while some viruses may contribute more strongly to a component based on prevalence or detectability, the influential viruses were expected to account for 55.1% of the explained variance at most, with the remaining weight still spread across other viruses providing complementary information.

The variance inflation factor (VIF) was calculated to detect multicollinearity in regression analysis, the VIF can estimate how much the variance of a regression coefficient is inflated due to multicollinearity. The higher the VIF, the higher the possibility that multicollinearity exists. When VIF is higher than 5, there is significant multicollinearity that needs to be corrected^34^. In our analysis, no variable with a VIF value greater than 5 was identified in the final model. We estimated the β coefficient and its 95% CI to describe the precision of the point estimate. A *P* value of < 0.05 was considered statistically significant and STATA 17.0 IC (Stata Corp LLC, College Station, TX) was used to analyze the data.

#### Mixed effect model

In our study, participants each had two repeated measurements taken, and measurements within individuals are likely correlated. We found results largely consistent with our original general linear models (Tables 2 and 3). Given our simple linear regression approach, more complex multilevel techniques risked overfitting unnecessary variance components and producing inflated estimates, whereas our priority was estimating the effects of main exposures of interest on child growth change. For the mixed effect model analysis, we used *R version 4.4.0*.

### Supplementary Information

Supplementary Information 1.

Supplementary Information 2.

Supplementary Information 3.

Supplementary Information 4.

Supplementary Information 5.

## Data Availability

A publicly available GEMS dataset was analyzed in this study. This data can be obtained here: ClinEpiDB (https://clinepidb.org/ce/app/workspace/analyses/DS_841a9f5259/new/variables/PCO_0000024/ENVO_00000009). Following the thorough review and approval process by the ClinEpiDB team, we have obtained official data access from ClinEpiDB, the responsible entity for managing the GEMS data repository.
