# Supplementary material for: Enteric viral pathogens and child growth among under-five children: findings from South Asia and sub-Saharan Africa
Source: Sci Rep. 2024 Jun 15;14:13871. doi: 10.1038/s41598-024-64374-0 (PMC11180137; doi:10.1038/s41598-024-64374-0)
Supplement: Supplementary file 1 — Supplementary Information 1. [file 41598_2024_64374_MOESM1_ESM.pdf]

Supplementary Table 1. Baseline characteristics of the symptomatic MSD and asymptomatic children having stool positive for viral pathogens (Rotavirus, norovirus, adenovirus, astrovirus, and sapovirus) in South Asia and sub-Saharan Africa (continued in the next table).

	Symptomatic			Asymptomatic			Symptomatic			Asymptomatic			Symptomatic			Asymptomatic		
**Characteristics**	**Rotavirus (+)**	**Rotavirus (-)**	**p-value**	**Rotavirus (+)**	**Rotavirus (-)**	**p-value**	**Norovirus (+)**	**Norovirus (-)**	**p-value**	**Norovirus (+)**	**Norovirus (-)**	**p-value**	**Adenovirus (+)**	**Adenovirus (-)**	**p-value**	**Adenovirus (+)**	**Adenovirus (-)**	**p-value**
n (%)	1747 (18.5)	7691 (81.5)		509(3.9)	12619 (96.1)		741(7.9)	8697 (92.2)		962(7.3)	12167 (92.7)		235(2.5)	9198 (97.5)		98(0.8)	13028 (99.3)	
**Age group**																		
0-11m	1,016 (58.2)	3013 (39.2)		245 (48.1)	4632 (36.7)		342 (46.2)	3687 (42.4)		367 (38.2)	4511 (37.1)		123 (52.3)	3904 (42.4)		41 (41.8)	4834 (37.1)	
12-23m	572 (32.7)	2632 (34.2)	<0.01	173 (33.9)	4208 (33.4)	0.01	253 (34.1)	2951 (33.9)	0.03	340 (35.3)	4041 (33.2)	0.10	89 (37.9)	3112 (33.8)	0.01	42 (42.9)	4339 (33.3)	0.01
24-59m	159 (9.1)	2046 (26.6)		91 (17.9)	3779 (29.9)		146(19.7)	2059 (23.7)		255 (26.5)	3615 (29.7)		23 (9.8)	2182 (23.7)		15 (15.3)	3855 (29.6)	
**Gender (**Girl)	804 (46.0)	3291 (42.8)	0.01	202 (39.7)	5448 (43.2)	0.12	322 (43.5)	3773 (43.4)	0.97	406 (42.2)	5245 (43.1)	0.59	114 (48.5)	3977 (43.2)	0.12	43 (43.9)	5606 (43.0)	0.87
**Anthropometry** (at enrollment)																		
HAZ[¥]	-1.13 ±1.37	- 1.38±1.36		- 1.38±1.30	-1.34± 1.31		-1.38±1.36	-1.33 ± 1.37		- 1.51±1.35	- 1.33±1.31		- 1.27±1.34	-1.34± 1.37		-1.49±1.47	-1.34± 1.31	
WAZ[¥]	- 1.44±1.37	-1.53± 1.4		- 1.00±1.28	-1.08± 1.32		-1.51±1.38	-1.51± 1.39		- 1.33±1.33	- 1.06±1.31		- 1.47±1.42	-1.51± 1.39		-1.26±1.59	-1.08± 1.31	
WHZ[¥]	- 1.09±1.50	- 1.06±1.49		- 0.32±1.44	-0.47± 1.43		-1.04±1.40	-1.07± 1.50		- 0.68±1.41	-0.45± 1.43		- 1.05±1.48	-1.06± 1.49		-0.63±1.79	-0.47± 1.43	
**Breastfeeding status**																		
Breastfeed	1521 (87.1)	5218 (67.9)		393 (77.2)	8645 (68.5)		545(73.6)	6194 (71.2)		661(68.7)	8378 (68.9)		203(86.4)	6531 (71)		75(76.5)	8961 (68.8)	
non-breastfed	226 (12.9)	2472 (32.2)	0.01	116 (22.8)	3974 (31.5)	0.01	196 (26.5)	2502 (28.8)	0.18	301 (31.3)	3789 (31.1)	0.92	32 13.6)	2666 (28.9)	0.01	23 (23.5)	4067 (31.2)	0.09
**Wealth quintile**																		
Poorest	380 (21.8)	1646 (21.4)		86(16.9)	2424 (19.2)		171(23.1)	1855 (21.3)		175(18.2)	2335 (19.2)		46(19.6)	1979 (21.5)		23(23.5)	2485 (19.1)	
Lower middle	310 (17.8)	1503 (19.6)		104(20.4)	2486 919.7)		136(18.4)	1677 (19.3)		194(20.2)	2396 (19.7)		44(18.7)	1768 (19.2)		18(18.4)	2572 (19.7)	
Middle	359 (20.6)	1634 (21.3)	0.32	110(21.6)	2724 921.6)	0.73	170(22.9)	1823 (20.9)	0.21	233(24.2)	2601 (21.4)	0.26	57(24.3)	1936 (21.1)	0.80	17(17.4)	2817 (21.6)	0.62
Upper middle	349 (19.9)	1430 (18.6)		105(20.6)	2417 (19.2)		142(19.2)	1637 (18.8)		179(18.6)	2343 (19.3)		42(17.9)	1735 (18.9)		22(22.5)	2500 (19.2)	
Richest	348 (19.9)	1473 (19.2)		104(20.4)	2567 (20.3)		122(16.5)	1699 (19.6)		181(18.8)	2491 (20.5)		46(19.6)	1774 (19.3)		18(18.4)	2653 (20.4)	
									**WASH**									
**The main source of drinking water**																		
Tube well water	275(15.7)	1400 (18.2)		100(19.65)	2894 (22.9)		132(17.8)	1543 (17.7)		195(20.3)	2799 (23)		44(18.7)	1631 (17.7)		25(25.5)	2967 (22.8)	
Non-tube well water	1472(84.3)	6291 (81.8)	0.02	409(80.4)	9725 (77.1)	0.08	609(82.2)	7154 (82.3)	0.96	767(79.7)	9368 (77)	0.05	191(81.3)	7567 (82.3)	0.70	73(74.5)	10061 (77.2)	0.52

**Toilet facility**																		
Sanitary/ semi sanitary	1675(95.9)	7302 (94.9)		482(94.7)	11806 (93.6)		701(94.6)	8276 (95.2)		905(94.1)	11384 (93.6)		227(96.6)	8745 (95.1)		91(92.9)	12195 (93.6)	
Non-sanitary	72(4.1)	389 (5.1)	0.10	27(5.3)	813 (6.4)	0.30	40(5.4)	421 (4.8)	0.49	57(5.9)	783 (6.4)	0.53	8(3.4)	453 (4.9)	0.29	7(7.1)	833 (6.4)	0.77
**Hand washing**																		
With soap and water	1304(74.7)	5825 (75.7)		356(70.2)	9405 (74.5)		536(72.3)	6593 (75.8)		718(74.6)	9044 (74.3)		172(73.2)	6953 (75.6)		69(70.4)	9692 (74.4)	
Without soap	442(25.3)	1866 (24.3)	0.36	151(29.8)	3214 (25.5)	0.03	205(27.7)	2103 (24.2)	0.03	244(25.4)	3121 (25.7)	0.84	63(26.8)	2244 (24.4)	0.40	29(29.6)	3334 (25.6)	0.37
**Clinical features**																		
Dysentery	129(7.4)	2108 (27.4)	0.01	-	-		215(29.0)	6675 (76.8)	0.01	-	-		26(11.06)	2211 (24.0)	0.01	-	-	
Fever	1093(62.6)	4,755 (61.8)	0.57	-	-		434(58.6)	5,414 (62.3)	0.05	-	-		143(60.9)	5,704 (62.0)	0.72	-	-	
Vomiting	1158(66.3)	2,487 (32.3)	0.01	-	-		271(36.6)	3,374 (38.8)	0.23	-	-		131(55.7)	3,514 (38.2)	0.01	-	-	

¶ mean± SD (standard deviation); height/length-for-age, weight-for-age, and weight-for-height/ length z-scores (HAZ/LAZ, WAZ, and WHZ); Breastfeed: partial and exclusive breastfeeding; Vomiting >=3 times per day

Continued in the next table

Supplementary table 1. Continued.

	Symptomatic			Asymptomatic			Symptomatic			Asymptomatic		
**Characteristics**	**Astrovirus (+)**	**Astrovirus (-)**	**p-value**	**Astrovirus (+)**	**Astrovirus (-)**	**p-value**	**Sapovirus (+)**	**Sapovirus (-)**	**p-value**	**Sapovirus (+)**	**Sapovirus (-)**	**p-value**
n (%)	238(2.5)	9200 (97.5)		261(1.9)	12868 (98.0)		325(3.4)	9113 (96.6)		456(3.5)	12673 (96.5)	
**Age group**												
0-11 Months	124 (52.1)	3905 (42.5)		103 (39.46)	4775 (37.1)		139 (42.77)	3890 (42.7)		178 (39.04)	4700 (37.1)	
12-23 Months	78 (32.77)	3126 (33.9)	0.01	84 (32.18)	4297 (33.4)	0.74	131 (40.31)	3073 (33.7)	0.01	173 (37.94)	4208 (33.2)	0.01
24-59 Months	36 (15.13)	2169 (23.6)		74 (28.35)	3796 (29.5)		55 (16.92)	2150 (23.6)		105 (23.03)	3765 (29.7)	
**Gender (**Girl)	102 (42.86)	3993 (43.4)	0.87	88 (33.72)	5563 (43.2)	0.01	123 (37.85)	3972 (43.6)	0.04	180 (39.47)	5471 (43.2)	0.12
**Anthropometry** (at enrollment)												
HAZ[¥]	-1.54±1.31	-1.33± 1.37		-1.44±1.37	-1.34±1.31		-1.38±1.40	-1.33± 1.36		-1.48±1.38	-1.34 ± 1.31	
WAZ[¥]	-1.69±1.39	-1.51± 1.39		-1.14±1.32	-1.08±1.32		-1.42±1.41	-1.51± 1.39		-1.30±1.34	-1.07± 1.32	
WHZ[¥]	-1.11±1.41	-1.06± 1.49		-0.48±1.44	-0.47± 1.43		-0.91±1.54	-1.07± 1.49		-0.70±1.36	-0.46± 1.43	
**Breastfeeding status**												
Breastfeed	176(73.95)	6563 (71.3)		172(65.9)	8867 (68.9)		244(75.08)	6495 (71.3)		330(72.37)	8709 (68.7)	
non-breastfed	62 (26.1)	2636 (86.7)	0.38	89 (34.1)	4001 (31.1)	0.30	81 (24.9)	2617 (28.7)	0.14	126 (27.6)	3964 (31.3)	0.10
**Wealth quintile**												
Poorest	53(22.27)	1973 (21.5)		50(19.16)	2460 (19.1)		64(19.75)	1962 (21.5)		73(16.01)	2437 (19.2)	
Lower middle	44(18.49)	1769 (19.2)		46(17.62)	2544 (19.8)		64(19.75)	1749 (19.2)		96(21.05)	2494 (19.7)	
Middle	49(20.6)	1944 (21.1)	0.99	61(23.37)	2773 (21.6)	0.89	78(24.1)	1915 (21)	0.70	107(23.46)	2727 (21.5)	0.40
Upper middle	47(19.8)	1732 (18.8)		49(18.77)	2473 (19.2)		57(17.6)	1722 (18.9)		92(20.18)	2430 (19.2)	
Richest	45(18.9)	1776 (19.3)		55(21.07)	2617 (20.3)		61(18.8)	1760 (19.3)		88(19.3)	2584 (20.4)	
**WASH**												
**The main source of drinking water**												
Tube well water	32(13.5)	1643 (17.9)		51(19.54)	2943 (22.9)		36(11.1)	1639 (17.9)		79(17.32)	2915 (23.0)	
Non-tube well water	206(86.6)	7557 (82.1)	0.08	210(80.46)	9925 (77.1)	0.20	289(88.9)	7474 (82.1)	0.001	377(82.7)	9758 (77.0)	0.01
**Toilet facility**												
Sanitary/ semi sanitary	229(96.2)	8748 (95.1)		248(95.02)	12041 (93.6)		305(93.9)	8672 (95.2)		435(95.4)	11854 (93.5)	
Non-sanitary	96.22(3.8)	452 (4.9)	0.42	13(4.98)	827 (6.4)	0.35	93.85(6.2)	441 (4.8)	0.28	21(4.6)	819 (6.5)	0.11
**Hand washing**												
With soap and water	169(71.01)	6960 (75.7)		179(68.58)	9583 (74.5)		242(74.5)	6887 (75.6)		335(73.5)	9427 (74.4)	
Without soap	69(28.99)	2239 (24.3)	0.10	82(31.42)	3283 (25.5)	0.031	83(25.5)	2225 (24.4)	0.64	121(26.5)	3244 (25.6)	0.65
**Clinical features**												
Dysentery	41(17.2)	2196 (23.9)	0.02	-	-		62(19.1)	2175 (23.9)	0.05	-	-	
Fever	149(62.6)	5,699 (61.9)	0.84	-	-		186(57.2)	5,662 (62.1)	0.07	-	-	
Vomiting	97(40.8)	3,548 (38.6)	0.49	-	-		130(40)	3,515 (38.6)	0.61	-	-	

[¶] mean± SD (standard deviation); height/length-for-age, weight-for-age, and weight-for-height/ length z-scores (HAZ/LAZ, WAZ, and WHZ); Breastfeed: partial and exclusive breastfeeding; Vomiting >=3 times per day
