# Supplementary material for: Enteric viral pathogens and child growth among under-five children: findings from South Asia and sub-Saharan Africa
Source: Sci Rep. 2024 Jun 15;14:13871. doi: 10.1038/s41598-024-64374-0 (PMC11180137; doi:10.1038/s41598-024-64374-0)
Supplement: Supplementary file 2 — Supplementary Information 2. [file 41598_2024_64374_MOESM2_ESM.pdf]

Supplementary Table 2: Association between enteric viral pathogens and child growth (Anthropometry: HAZ/LAZ, WAZ, and WHZ): results of multiple linear regression modeling and mixed effect modeling (dependent variables— HAZ/LAZ, WAZ, and WHZ) among under-five children in South Asia and sub-Saharan Africa
Comparison between mixed effect model and multiple linear regression model

Symptomatic MSD children

		Mixed effect model			Multiple linear regression					
	Predictors	***Estimates***	***CI***	***p***	***Estimates***	***CI***	***p***	**Random Effects**		
**HAZ**	Rotavirus	**0.11**	**0.04 – 0.18**	**0.001**	**0.11**	**0.06 – 0.16**	**<0.001**	σ^2	0.2	
	Norovirus	0.09	-0.01 – 0.18	0.081	**0.08**	**0.01 – 0.15**	**0.031**	τ_{00}	1.32 $_{childid}$	
	Adenovirus	-0.06	-0.22 – 0.11	0.499	-0.05	-0.17 – 0.07	0.392	Marginal R^2 / Conditional R^2	0.136 / 0.885	0.135
	Astrovirus	-0.16	-0.32 – 0.01	0.062	**-0.16**	**-0.28 – -0.04**	**0.008**			
	Sapovirus	0.03	-0.11 – 0.18	0.633	0.02	-0.08 – 0.13	0.665			
**WAZ**	Rotavirus	0.04	-0.02 – 0.11	0.202	0.04	-0.01 – 0.09	0.125	σ^2	0.42	
	Norovirus	**0.14**	**0.04 – 0.24**	**0.005**	**0.13**	**0.06 – 0.21**	**<0.001**	τ_{00}	1.26 $_{childid}$	
	Adenovirus	0	-0.17 – 0.17	0.999	0.01	-0.12 – 0.13	0.911	Marginal R^2 / Conditional R^2	0.107 / 0.774	0.109
	Astrovirus	-0.09	-0.26 – 0.08	0.294	-0.09	-0.22 – 0.04	0.158			
	Sapovirus	0.13	-0.02 – 0.27	0.08	**0.12**	**0.02 – 0.23**	**0.024**			
**WHZ**	Rotavirus	-0.04	-0.11 – 0.03	0.293	-0.04	-0.10 – 0.01	0.144	σ^2	0.91	
	Norovirus	**0.13**	**0.03 – 0.22**	**0.012**	**0.12**	**0.04 – 0.20**	**0.003**	τ_{00}	1.04 $_{childid}$	
	Adenovirus	0.03	-0.14 – 0.20	0.75	0.03	-0.10 – 0.17	0.645	Marginal R^2 / Conditional R^2	0.088 / 0.576	0.09
	Astrovirus	0.001	-0.17 – 0.17	0.995	0.001	-0.13 – 0.14	0.944			
	Sapovirus	**0.16**	**0.01 – 0.30**	**0.031**	**0.16**	**0.04 – 0.27**	**0.009**			

Asymptomatic children

		Mixed effect model			Multiple linear regression					
	Predictors	***Estimates***	***CI***	***p***	***Estimates***	***CI***	***p***			
**HAZ**	Rotavirus	-0.04	-0.15 – 0.07	0.469	-0.04	-0.12 – 0.04	0.311	σ^2	0.22	
	Norovirus	-0.05	-0.14 – 0.03	0.183	-0.05	-0.11 – 0.01	0.105	τ_{00}	1.28 $_{childid}$	
	Adenovirus	-0.12	-0.36 – 0.13	0.364	-0.12	-0.30 – 0.07	0.215	Marginal R^2 / Conditional R^2	0.128 / 0.872	0.129
	Astrovirus	-0.04	-0.19 – 0.11	0.611	-0.04	-0.15 – 0.07	0.493			
	Sapovirus	-0.06	-0.17 – 0.06	0.339	-0.06	-0.15 – 0.02	0.162			
**WAZ**	Rotavirus	-0.09	-0.20 – 0.02	0.093	-0.09	-0.18 – -0.01	**0.026**	σ^2	0.37	
	Norovirus	**-0.13**	**-0.21 – -0.05**	**0.001**	**-0.12**	**-0.18 – -0.06**	**<0.001**	τ_{00}	1.14 $_{childid}$	
	Adenovirus	**-0.27**	**-0.52 – -0.03**	**0.027**	**-0.27**	**-0.46 – -0.09**	**0.003**	Marginal R^2 / Conditional R^2	0.115 / 0.782	0.115
	Astrovirus	0.02	-0.13 – 0.17	0.799	0.02	-0.09 – 0.13	0.735			
	Sapovirus	**-0.14**	**-0.25 – -0.02**	**0.017**	**-0.14**	**-0.23 – -0.06**	**0.001**			
**WHZ**	Rotavirus	-0.1	-0.21 – 0.01	0.076	**-0.1**	**-0.19 – -0.01**	**0.025**	σ^2	0.76	
	Norovirus	**-0.13**	**-0.22 – -0.05**	**0.001**	**-0.13**	**-0.20 – -0.07**	**<0.001**	τ_{00}	1.03 $_{childid}$	
	Adenovirus	**-0.34**	**-0.59 – -0.09**	**0.008**	**-0.34**	**-0.54 – -0.14**	**0.001**	Marginal R^2 / Conditional R^2	0.091 / 0.615	0.091
	Astrovirus	0.04	-0.11 – 0.19	0.606	0.04	-0.08 – 0.16	0.515			
	Sapovirus	**-0.17**	**-0.29 – -0.06**	**0.004**	**-0.18**	**-0.27 – -0.08**	**<0.001**			

Presence of any viral pathogen

	Mixed effect model			Multiple linear regression			Random effect		
**Symptomatic MSD Children**	***Estimates***	***CI***	***p***	***Estimates***	***CI***	***p***	τ_{00}	Marginal R^2 / Conditional R^2	
**HAZ**	**0.09**	**0.03 – 0.14**	**0.003**	**0.08**	**0.04 – 0.12**	**<0.001**	1.32	0.136 / 0.885	0.135
**WAZ**	**0.07**	**0.01 – 0.13**	**0.016**	**0.07**	**0.02 – 0.11**	**0.003**	1.26	0.106 / 0.774	0.108
**WHZ**	0.02	-0.04 – 0.08	0.506	0.02	-0.03 – 0.06	0.504	1.04	0.106 / 0.774	0.108
**Asymptomatic Children**									
**HAZ**	-0.05	-0.11 – 0.01	0.1	**-0.05**	**-0.09 – -0.00**	**0.032**	1.28	0.128 / 0.872	0.129
**WAZ**	**-0.12**	**-0.18 – -0.06**	**<0.001**	**-0.12**	**-0.16 – -0.07**	**<0.001**	1.14	0.116 / 0.782	0.116
**WHZ**	**-0.14**	**-0.20 – -0.08**	**<0.001**	**-0.14**	**-0.18 – -0.09**	**<0.001**	1.03	0.092 / 0.615	0.092

Combined viral pathogen

**Asymptomatic Children**	***Estimates***	***CI***	***p***	***Estimates***	***CI***	***p***			
**HAZ**	-0.04	-0.11 – 0.02	0.194	-0.04	-0.09 – 0.01	0.1	1.28	0.128 / 0.872	0.129
**WAZ**	**-0.11**	**-0.17 – -0.04**	**0.001**	**-0.1**	**-0.15 – -0.06**	**<0.001**	1.14	0.116 / 0.782	0.116
**WHZ**	**-0.12**	**-0.19 – -0.06**	**<0.001**	**-0.12**	**-0.17 – -0.07**	**<0.001**	1.03	0.092 / 0.615	0.092
**Symptomatic MSD Children**									
**HAZ**	0.02	-0.06 – 0.09	0.708	0.01	-0.05 – 0.06	0.817	1.32	0.135 / 0.885	0.134
**WAZ**	0.06	-0.02 – 0.14	0.146	0.05	-0.01 – 0.12	0.079	1.26	0.106 / 0.774	0.108
**WHZ**	0.07	-0.01 – 0.15	0.086	**0.07**	**0.00 – 0.14**	**0.037**	1.04	0.088 / 0.576	0.09
