# Supplementary material for: Enteric viral pathogens and child growth among under-five children: findings from South Asia and sub-Saharan Africa
Source: Sci Rep. 2024 Jun 15;14:13871. doi: 10.1038/s41598-024-64374-0 (PMC11180137; doi:10.1038/s41598-024-64374-0)
Supplement: Supplementary file 3 — Supplementary Information 3. [file 41598_2024_64374_MOESM3_ESM.pdf]

Supplementary Table 3: Association between enteric viral pathogens and child growth (Anthropometry: HAZ/LAZ, WAZ, and WHZ): results of multiple linear regression modeling and mixed effect model (dependent variables— HAZ/LAZ, WAZ, and WHZ) among the different age groups in South Asia and sub-Saharan Africa

**HAZ**	**Mixed effect model**			**Multiple linear regression**		
Predictors	***Estimates***	***CI***	***p***	***Estimates***	***CI***	***p***
**Symptomatic MSD children**						
**Rotavirus**						
0-11 months	**0.1**	**0.01 – 0.19**	**0.033**	**0.11**	**0.05 – 0.18**	**0.001**
11-23 months	**0.16**	**0.04 – 0.28**	**0.01**	**0.16**	**0.07 – 0.25**	**<0.001**
24-59 months	0.05	-0.15 – 0.26	0.607	0.06	-0.08 – 0.21	0.392
**Norovirus**						
0-11 months	0.1	-0.04 – 0.24	0.181	0.09	-0.01 – 0.20	0.085
11-23 months	0.06	-0.10 – 0.23	0.468	0.05	-0.07 – 0.17	0.389
24-59 months	0.08	-0.13 – 0.29	0.475	0.08	-0.07 – 0.23	0.315
**Adenovirus**						
0-11 months	-0.06	-0.28 – 0.17	0.629	-0.03	-0.19 – 0.14	0.727
11-23 months	-0.09	-0.36 – 0.19	0.535	-0.08	-0.28 – 0.12	0.418
24-59 months	0.08	-0.41 – 0.56	0.761	0.08	-0.27 – 0.43	0.659
**Astrovirus**						
0-11 months	-0.23	-0.45 – 0.00	0.052	**-0.24**	**-0.41 – -0.07**	**0.005**
11-23 months	0.03	-0.26 – 0.33	0.815	0.04	-0.17 – 0.25	0.708
24-59 months	-0.37	-0.78 – 0.04	0.077	**-0.37**	**-0.67 – -0.08**	**0.014**
**Sapovirus**						
0-11 months	0.03	-0.18 – 0.24	0.766	0.01	-0.15 – 0.16	0.92
11-23 months	0.02	-0.21 – 0.25	0.863	0.02	-0.15 – 0.19	0.82
24-59 months	0.13	-0.20 – 0.47	0.43	0.13	-0.11 – 0.37	0.287
	**Mixed effect model**			**Multiple linear regression**		
Predictors	***Estimates***	***CI***	***p***	***Estimates***	***CI***	***p***
**Asymptomatic Children**						
**Rotavirus**						
0-11 months	**-0.19**	**-0.35 – -0.03**	**0.023**	**-0.19**	**-0.31 – -0.07**	**0.002**
11-23 months	0.01	-0.19 – 0.21	0.918	0.01	-0.14 – 0.15	0.922
24-59 months	0.23	-0.01 – 0.47	0.057	**0.23**	**0.06 – 0.40**	**0.008**
**Norovirus**						
0-11 months	-0.07	-0.20 – 0.07	0.319	-0.06	-0.16 – 0.04	0.259
11-23 months	-0.05	-0.19 – 0.09	0.481	-0.05	-0.15 – 0.05	0.321
24-59 months	-0.06	-0.21 – 0.08	0.399	-0.05	-0.16 – 0.05	0.309
**Adenovirus**						
0-11 months	0.05	-0.34 – 0.44	0.81	0.05	-0.24 – 0.34	0.745
11-23 months	-0.32	-0.71 – 0.07	0.108	**-0.32**	**-0.61 – -0.04**	**0.028**
24-59 months	0.001	-0.59 – 0.59	0.995	0	-0.42 – 0.42	0.993
**Astrovirus**						
0-11 months	0.02	-0.23 – 0.27	0.858	0.02	-0.16 – 0.21	0.807
11-23 months	-0.24	-0.51 – 0.04	0.088	**-0.24**	**-0.44 – -0.04**	**0.019**
24-59 months	0.12	-0.15 – 0.38	0.39	0.12	-0.07 – 0.31	0.232
**Sapovirus**						
0-11 months	0.07	-0.12 – 0.26	0.442	0.07	-0.07 – 0.21	0.346
11-23 months	-0.04	-0.23 – 0.15	0.686	-0.04	-0.18 – 0.10	0.56
24-59 months	**-0.34**	**-0.56 – -0.11**	**0.004**	**-0.34**	**-0.50 – -0.18**	**<0.001**
