# Supplementary material for: Enteric viral pathogens and child growth among under-five children: findings from South Asia and sub-Saharan Africa
Source: Sci Rep. 2024 Jun 15;14:13871. doi: 10.1038/s41598-024-64374-0 (PMC11180137; doi:10.1038/s41598-024-64374-0)
Supplement: Supplementary file 4 — Supplementary Information 4. [file 41598_2024_64374_MOESM4_ESM.pdf]

Supplementary Table 4: Association between enteric viral pathogens and child growth (Anthropometry: HAZ/LAZ, WAZ, and WHZ): results of multiple linear regression modeling and mixed effect model (dependent variables— HAZ/LAZ, WAZ, and WHZ) among the different age groups in South Asia and sub-Saharan Africa

**WAZ**	**Mixed effect model**			**Multiple linear regression**		
Predictors	***Estimates***	***CI***	***p***	***Estimates***	***CI***	***p***
**Symptomatic MSD children**						
**Rotavirus**						
0-11 months	0.02	-0.08 – 0.12	0.669	0.02	-0.06 – 0.10	0.624
12-23 months	0.11	-0.01 – 0.23	0.065	**0.11**	**0.02 – 0.20**	**0.02**
24-59 months	0.03	-0.15 – 0.21	0.717	0.03	-0.10 – 0.16	0.629
**Norovirus**						
0-11 months	**0.2**	**0.05 – 0.36**	**0.01**	**0.19**	**0.08 – 0.31**	**0.001**
12-23 months	0.11	-0.06 – 0.27	0.209	0.1	-0.02 – 0.23	0.106
24-59 months	0.07	-0.11 – 0.26	0.45	0.06	-0.07 – 0.20	0.373
**Adenovirus**						
0-11 months	0.05	-0.20 – 0.29	0.704	0.06	-0.13 – 0.25	0.526
12-23 months	-0.09	-0.36 – 0.19	0.538	-0.08	-0.29 – 0.12	0.418
24-59 months	0.05	-0.39 – 0.48	0.829	0.04	-0.27 – 0.35	0.8
**Astrovirus**						
0-11 months	-0.19	-0.43 – 0.06	0.142	**-0.2**	**-0.39 – -0.01**	**0.042**
12-23 months	0.12	-0.17 – 0.41	0.428	0.12	-0.10 – 0.34	0.278
24-59 months	-0.26	-0.63 – 0.10	0.157	-0.24	-0.51 – 0.03	0.078
**Sapovirus**						
0-11 months	0.16	-0.07 – 0.39	0.183	0.14	-0.03 – 0.32	0.11
12-23 months	0.12	-0.11 – 0.35	0.319	0.12	-0.05 – 0.30	0.157
24-59 months	0.16	-0.14 – 0.45	0.308	0.14	-0.07 – 0.36	0.19

	**Mixed effect model**			**Multiple linear regression**		
Predictors	***Estimates***	***CI***	***p***	***Estimates***	***CI***	***p***
**Asymtomatic**						
**Rotavirus**						
0-11 months	**-0.22**	**-0.39 – -0.06**	**0.009**	**-0.22**	**-0.35 – -0.09**	**0.001**
12-23 months	-0.14	-0.33 – 0.06	0.176	-0.14	-0.29 – 0.01	0.072
24-59 months	**0.29**	**0.07 – 0.50**	**0.009**	**0.28**	**0.13 – 0.44**	**<0.001**
**Norovirus**						
0-11 months	**-0.22**	**-0.36 – -0.09**	**0.002**	**-0.22**	**-0.32 – -0.11**	**<0.001**
12-23 months	-0.08	-0.22 – 0.06	0.255	-0.07	-0.18 – 0.03	0.175
24-59 months	-0.08	-0.21 – 0.05	0.238	-0.07	-0.17 – 0.02	0.141
**Adenovirus**						
0-11 months	-0.29	-0.69 – 0.11	0.161	-0.29	-0.59 – 0.02	0.068
12-23 months	-0.32	-0.71 – 0.07	0.11	**-0.32**	**-0.61 – -0.02**	**0.035**
24-59 months	-0.07	-0.60 – 0.45	0.785	-0.07	-0.45 – 0.31	0.708
**Astrovirus**						
0-11 months	-0.02	-0.28 – 0.23	0.853	-0.02	-0.22 – 0.17	0.821
12-23 months	-0.03	-0.30 – 0.25	0.854	-0.03	-0.23 – 0.18	0.799
24-59 months	0.13	-0.11 – 0.37	0.285	0.13	-0.04 – 0.30	0.143
**Sapovirus**						
0-11 months	-0.03	-0.22 – 0.17	0.8	-0.03	-0.18 – 0.12	0.663
12-23 months	-0.12	-0.31 – 0.07	0.23	-0.12	-0.26 – 0.03	0.108
24-59 months	**-0.39**	**-0.59 – -0.19**	**<0.001**	**-0.39**	**-0.54 – -0.25**	**<0.001**
