# Supplementary material for: Enteric viral pathogens and child growth among under-five children: findings from South Asia and sub-Saharan Africa
Source: Sci Rep. 2024 Jun 15;14:13871. doi: 10.1038/s41598-024-64374-0 (PMC11180137; doi:10.1038/s41598-024-64374-0)
Supplement: Supplementary file 5 — Supplementary Information 5. [file 41598_2024_64374_MOESM5_ESM.pdf]

Supplementary Table 5: Association between enteric viral pathogens and child growth (Anthropometry: HAZ/LAZ, WAZ, and WHZ): results of multiple linear regression modeling and mixed effect model (dependent variables— HAZ/LAZ, WAZ, and WHZ) among the different age groups in South Asia and sub-Saharan Africa

**WHZ**	**Mixed effect model**			**Multiple linear regression**		
**Symptomatic MSD Children**						
Predictors	***Estimates***	***CI***	***p***	***Estimates***	***CI***	***p***
**Rotavirus**						
0-11 months	-0.09	-0.19 – 0.01	0.091	**-0.09**	**-0.18 – -0.01**	**0.035**
12-23 months	0.05	-0.07 – 0.17	0.402	0.05	-0.05 – 0.14	0.332
24-59 months	-0.01	-0.18 – 0.17	0.931	-0.02	-0.16 – 0.11	0.756
**Norovirus**						
0-11 months	**0.18**	**0.02 – 0.34**	**0.024**	**0.18**	**0.05 – 0.31**	**0.006**
12-23 months	0.11	-0.05 – 0.28	0.184	0.11	-0.02 – 0.24	0.103
24-59 months	0.07	-0.12 – 0.25	0.48	0.05	-0.09 – 0.19	0.458
**Adenovirus**						
0-11 months	0.08	-0.17 – 0.34	0.519	0.09	-0.12 – 0.30	0.391
12-23 months	-0.06	-0.33 – 0.21	0.657	-0.06	-0.28 – 0.16	0.596
24-59 months	0.02	-0.41 – 0.44	0.935	0.01	-0.32 – 0.34	0.946
**Astrovirus**						
0-11 months	-0.11	-0.37 – 0.15	0.409	-0.11	-0.32 – 0.10	0.32
12-23 months	0.15	-0.15 – 0.44	0.328	0.14	-0.09 – 0.38	0.224
24-59 months	-0.04	-0.39 – 0.32	0.847	-0.01	-0.29 – 0.27	0.952
**Sapovirus**						
0-11 months	0.18	-0.06 – 0.42	0.144	0.17	-0.03 – 0.37	0.094
12-23 months	0.15	-0.08 – 0.38	0.2	0.16	-0.03 – 0.34	0.095
24-59 months	0.15	-0.14 – 0.45	0.301	0.14	-0.09 – 0.37	0.229
**Asymptomatic children**						
**Rotavirus**						
0-11 months	**-0.19**	**-0.37 – -0.01**	**0.035**	**-0.19**	**-0.33 – -0.04**	**0.011**
12-23 months	-0.18	-0.38 – 0.02	0.082	**-0.19**	**-0.35 – -0.02**	**0.025**
24-59 months	**0.23**	**0.02 – 0.44**	**0.031**	**0.23**	**0.07 – 0.38**	**0.005**
**Norovirus**						
0-11 months	**-0.27**	**-0.41 – -0.12**	**<0.001**	**-0.27**	**-0.39 – -0.15**	**<0.001**
12-23 months	-0.07	-0.21 – 0.07	0.35	-0.06	-0.17 – 0.05	0.308
24-59 months	-0.06	-0.19 – 0.07	0.402	-0.05	-0.15 – 0.05	0.323
**Adenovirus**						
0-11 months	**-0.48**	**-0.90 – -0.05**	**0.027**	**-0.48**	**-0.82 – -0.13**	**0.006**
12-23 months	-0.24	-0.63 – 0.16	0.245	-0.24	-0.56 – 0.08	0.149
24-59 months	-0.13	-0.65 – 0.38	0.618	-0.13	-0.52 – 0.26	0.507
**Astrovirus**						
0-11 months	-0.09	-0.36 – 0.18	0.534	-0.08	-0.30 – 0.13	0.449
12-23 months	0.13	-0.15 – 0.40	0.374	0.13	-0.10 – 0.35	0.27
24-59 months	0.1	-0.14 – 0.33	0.421	0.09	-0.08 – 0.27	0.29
**Sapovirus**						
0-11 months	-0.13	-0.34 – 0.07	0.197	-0.14	-0.31 – 0.03	0.099
12-23 months	-0.15	-0.34 – 0.05	0.143	-0.15	-0.30 – 0.01	0.068
24-59 months	**-0.29**	**-0.49 – -0.09**	**0.005**	**-0.28**	**-0.43 – -0.13**	**<0.001**
